# Profound Elevation in LDL Cholesterol Level Following a Ketogenic Diet: A Case Series

**DOI:** 10.1016/j.cjco.2022.05.001

**Published:** 2022-06-09

**Authors:** Rebecca Crosier, Ruth McPherson

**Affiliations:** a Department of Medicine, University of Ottawa, Ottawa, Ontario, Canada; b University of Ottawa Heart Institute, University of Ottawa, Ottawa, Ontario, Canada; c Division of Cardiology, Department of Medicine, University of Ottawa, Ottawa, Ontario, Canada

## Abstract

The ketogenic diet (KD) is currently popular for the achievement of weight loss and improvement in glycemic variables. The diet allows consumption of foods high in fat and protein, with strict limitation of carbohydrates. We present a case series of substantial increases in total cholesterol and low-density lipoprotein cholesterol following the initiation of a KD, with improvements in cholesterol levels once the KD was stopped. Novel teaching points include the need for lipid monitoring in patients who choose to follow a KD and for raising awareness of the extreme lipid response that can occur in some patients, particularly lean individuals.

## Résumé;

À l’heure actuelle, le régime cétogène (RC) est populaire pour obtenir une perte de poids et améliorer les variables de la glycémie. Le régime repose sur la consommation d’aliments riches en matières grasses et en protéines, et une stricte limitation des aliments riches en glucides. Nous présentons une série de cas dont le cholestérol total et le cholestérol à lipoprotéines de faible densité avaient augmenté de façon substantielle après l’adoption du RC, et dont les concentrations de cholestérol s’étaient améliorées après l’arrêt du RC. Parmi les nouveaux points à retenir figurent la nécessité d’effectuer le bilan lipidique chez les patients qui choisissent de suivre un RC et la conscientisation à la réponse lipidique extrême que l’on peut observer chez certains patients, particulièrement chez les personnes minces.

The ketogenic diet (KD), consisting of foods that are high in fat and cholesterol, with marked restriction of carbohydrate intake, has become a popular diet in Canada. The reduction in carbohydrate intake results in synthesis of ketones, which are used as fuel. Meta-analyses have shown that a KD can be effective for weight reduction. A meta-analysis of 15 studies concluded that very low-calorie ketone diets result in significant weight loss in the short term (–7.48 kg at 1 month), intermediate term (–16.76 kg at 4-6 months), and long term (–21.48 kg at 12 months).^1^

KDs may have favourable metabolic effects in diabetic patients, including lower hemoglobin (Hb)A1c and fasting glucose levels. However, systematic reviews and meta-analyses of randomized control trials have reported conflicting results on the effect of KDs on total cholesterol (TC) and low-density lipoprotein cholesterol (LDL-C) levels.^2^ Some studies demonstrate a reduction in LDL-C, whereas others report an increase, and others, no change. Despite conflicting findings, the changes in LDL-C level observed were generally small. In this report, we describe 3 cases in which LDL-C increased markedly in response to a KD. Key metabolic parameters for each case are outlined in Table 1.

**Table 1:** Summary of metabolic variables before, during, and after following a ketogenic diet (KD)

Variable	Case 1			Case 2			Case 3		
	Baseline	KD	Post	Baseline	KD	Post	Baseline	KD	Post
LDL-C	1.84	8.05	3.09	3.63	14.55	1.6	3.06	7.58	4.96
TC	3.91	10.9	6.39	5.94	16.58	3.7	6.06	10.76	8.08
HDL-C	1.87	2.4	2.99	1.92	1.62	1.84	2.52	2.60	2.67
TGs	0.43	0.99	0.68	0.86	0.90	0.59	1.04	1.27	0.98
HbA1c, %	5.1	N/A	4.9	5.3	5.0	5.4	5.9	5.9	6.1
Fasting glucose	4.5	4.3	4.7	5.7	4.9	5.2	4.8	5.1	4.5

All variables, excluding hemoglobin (Hb)A1c, are expressed in mmol/L.

HDL-C, high-density lipoprotein cholesterol; LDL-C, low-density lipoprotein cholesterol; N/A, not available; TC, total cholesterol; TGs, triglycerides.

## Case 1

**Figure 1:**
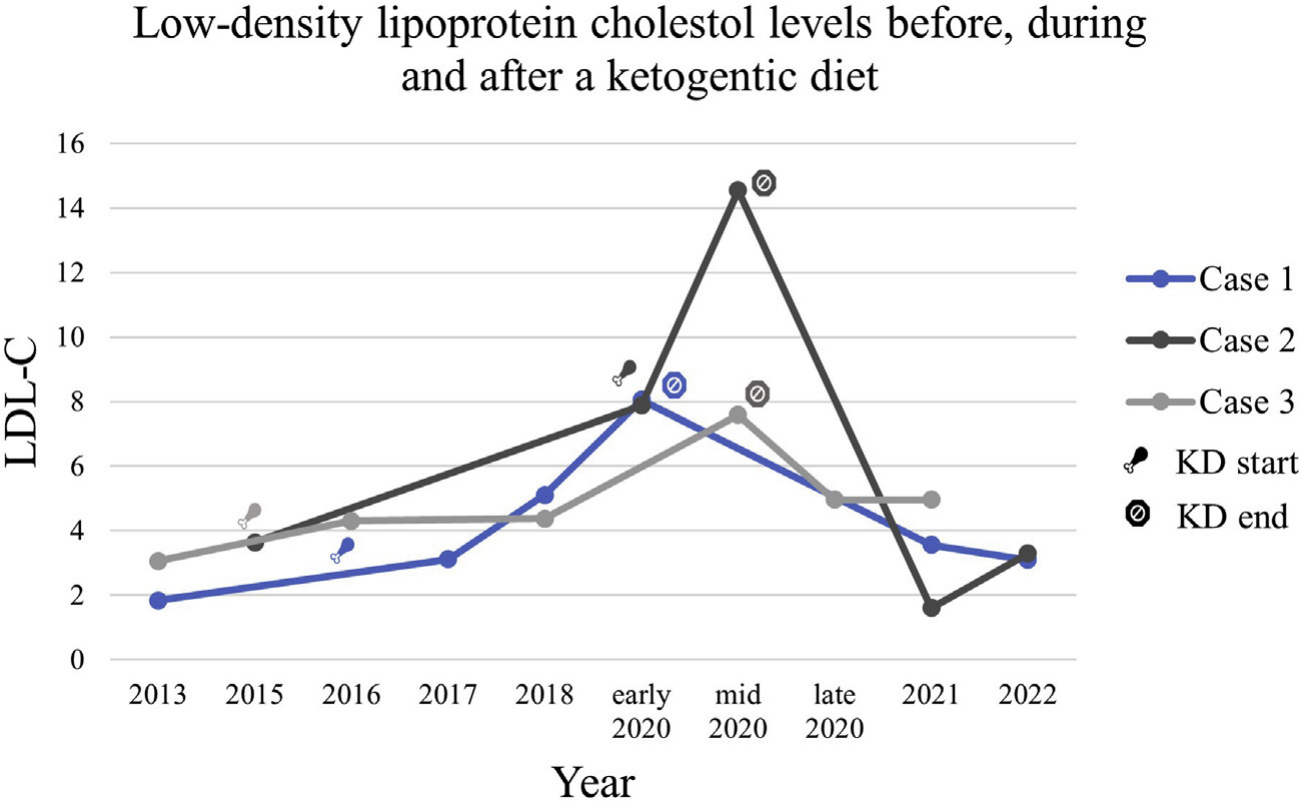
Low-density lipoprotein cholesterol (LDL-C) levels over time for each case, before, during, and after following a ketogenic diet (KD).

A 46-year-old premenopausal woman was referred for evaluation of severe dyslipidemia. She had a family history of hypercholesterolemia. She exercised daily and did not take any medications. She had a body mass index (BMI) of 20 kg/m^2^ and a normal physical examination. Her thyroid-stimulating hormone (TSH), creatinine, HbA1c, liver enzymes, and bilirubin levels were within normal limits. She began following a KD, with high consumption of red meats, butter, and cheese, and 2 eggs per day, with intermittent periods of consuming nothing but meat, 4 years prior to referral. While on this diet, more than 25% of her daily calories were from saturated fat. Cholesterol intake was approximately 400 to 600 mg/d. Of note, her body weight was unchanged during her diet. The trend of her LDL-C level over time is provided in Figure 1. Prior to initiating a KD, her LDL-C level was 1.84 mmol/L. After 4 years of following a KD, her LDL level was 8.05 mmol/L. She was then instructed to modify her diet, favouring vegetables, rice, and lean sources of protein and limiting egg yolks to 3/wk. The percentage of calories from saturated fat was reduced to 5%, and cholesterol intake was reduced to 250 mg/d. Her LDL-C level 1 year after dietary adjustment was 3.09 mmol/L.

## Case 2

A 57-year-old man was referred for evaluation of dyslipidemia. He had a history of chronic hepatitis B virus infection, for which he was taking tenofovir. His TSH, creatinine, HbA1c, and bilirubin levels, and urine albumin-creatinine ratio were within normal limits. Hi alanine aminotransferase level was mildly elevated at 60 U/L. He started following a KD, consisting largely of red meat, and 4 eggs per day. His BMI was 21.9 kg/m^2^ and did not change during 3 months on a KD. While he was on this diet, approximately 20% of his daily calories came from saturated fats, and his cholesterol intake was estimated to be greater than 1200 mg/d. His laboratory investigations well before initiating the KD demonstrated an LDL-C level of 3.63 mmol/L. After following a KD and starting tenofovir, his LDL-C level increased to 14.55 mmol/L. This rise was far greater than that expected with tenofovir alone. He was started on atorvastatin, 80 mg daily, with ezetimibe at 10 mg daily, and instructed to discontinue his KD. He continued taking tenofovir. He modified his diet, cutting out eggs, favouring lean meats and canola oil, and no longer restricting carbohydrates. The percentage of calories from saturated fats decreased to approximately 7%, and his cholesterol intake decreased to less than 200 mg/d. Seven months later, his total cholesterol level was 3.7 mmol/L, with an LDL-C level of 1.6 mmol/L (Fig. 1).

## Case 3

A 64-year-old woman was evaluated for severe hypercholesterolemia. Her clinical history included well controlled hypothyroidism (TSH = 1.97 mL U/L) and depression. Her creatinine, HbA1c, liver enzyme, and bilirubin levels were within normal limits. Her BMI was 21.5 kg/m^2^. She had been following a KD, with high consumption of butter, fatty meats, and coconut oil, for 5 years. Approximately 18% of her daily calories were from saturated fat. Her weight did not change during this period. Her LDL-C level prior to initiating a KD was 3.06 mmol/L. After 5 years of a KD, her LDL-C level was 7.58 mmol/L. Only 2 months after switching to a regular diet, with approximately 5% of daily calories from saturated fats, her LDL-C level was 4.54 mmol/L. This level was sustained 1 year later (Fig. 1).

## Discussion

Here we present 3 cases of normal-weight adults who experienced profound elevations in LDL-C level in response to a KD, with little to no change in trigylceride or glucose levels. In all cases, LDL-C level improved after cessation of the diet. In Case 3, just 2 months after stopping the KD, LDL-C level decreased by 40%. Likewise, in Case 2, 7 months after stopping the KD, LDL-C level had plummeted by 79%, a far greater drop than expected from medications alone.

Low-carbohydrate, high-fat diets have been used for the purposes of weight loss for decades. Recently, the KD, a low-carbohydrate/high-fat diet, has become widely popular for not just those wanting to lose weight, but also healthy normal-weight people. Restricting carbohydrates generally results in a high consumption of saturated fats and cholesterol. A causal link has been established between saturated-fat intake and LDL-C levels, and elevated LDL-C is a well-established risk factor for coronary atherosclerosis. In the literature, data are conflicting regarding LDL-C-level response to KDs. Some of the differences observed in the trials on effects on LDL-C level may reflect variations in carbohydrate and fat quantity and quality in the individual dietary interventions, differences in adherence, and differing amounts of weight loss, a factor that can itself result in lowering LDL-C level.

A study by Retterstøl et al. of young, normal-weight adults noted an average increase in LDL-C of 44% after 3 weeks of following a KD, compared with control patients, who had marked interindividual variation (5% to 107% increase in LDL-C).^3^ Another case series by Goldberg et al.^4^ included genetic testing on 4 patients who had large increases in LDL-C while on a KD. One person had an *APOE* E2/E2 genotype, 2 had a higher burden of common genetic polymorphisms that contribute to hyperlipidemia, and 1 did not have any identified genetic contributors. KDs are also high in cholesterol, in part dependent on egg-yolk consumption. As we reported previously, consumption of 3 eggs per day by young healthy men under controlled dietary conditions resulted in a mean 21.2% increase in LDL-C, with individual increases ranging from 0% to 62%.^5^

A point of note is that the 3 individuals described here were of normal body weight and did not experience weight loss. Thus, the composition of fatty acids reaching the liver reflected largely that of dietary intake. In contrast, during weight loss, as in obese individuals consuming a KD diet, a substantial contribution of fatty acids is derived from adipose tissue lipolysis, such that the proportion of saturated to unsaturated fatty acids available for hepatic metabolism is lower than it is during weight maintenance. This fact may account for the attenuated increase in LDL-C that occurs when a KD diet is prescribed for weight loss.

Multiple common genetic variants in genes encoding apolipoproteins and cholesterol transporters have been linked to interindividual responses to changes in dietary fat content and composition and cholesterol intake.^6^ The hepatic response to saturated fat intake is mediated through sterol-responsive element binding protein (SREBP), with downstream effects on lipogenic enzyme activity and LDL receptor number.^7^ In the Retterstøl study,^3^ participants in the low-carbohydrate group demonstrated a 30% decrease in LDL receptor expression compared to baseline.

In conclusion, the KD can be an effective diet for weight loss and may improve the glycemic variables and plasma triglycerides in obese individuals. As shown here, recognizing that a KD can elicit an extreme elevation in LDL-C level, particularly in lean, weight-stable individuals, is important. Per the American Heart Association guidelines, an optimal diet for cardiovascular disease prevention is one low in saturated fat and cholesterol, with inclusion of appropriate sources of polyunsaturated and monosaturated fats.^8^

**Novel Teaching Points**

• A KD can result in marked increases in LDL-C, particularly in lean, weight-stable individuals.
• A complex interplay of genetic factors may contribute to the variability of interindividual response to dietary saturated fat and cholesterol.
• Although a KD may have a role in eliciting short-term weight loss and improved glycemic control in obese individuals, it should not be recommended for weight maintenance.
